# ^19^F NMR and DFT Analysis Reveal Structural and Electronic Transition State Features for RhoA‐Catalyzed GTP Hydrolysis

**DOI:** 10.1002/anie.201509477

**Published:** 2016-01-28

**Authors:** Yi Jin, Robert W. Molt, Jonathan P. Waltho, Nigel G. J. Richards, G. Michael Blackburn

**Affiliations:** ^1^ Krebs Institute, Department of Molecular Biology and BiotechnologyUniversity of SheffieldSheffieldS10 2TNUK; ^2^ Department of Chemistry and Chemical BiologyIndiana University Purdue University IndianapolisIndianapolisIN46202USA; ^3^ Manchester Institute of BiotechnologyManchesterM1 7DNUK; ^4^ School of ChemistryCardiff UniversityCardiffCF10 3ATUK

**Keywords:** ^19^F NMR, enzyme catalysis, GTP hydrolases, phosphoryl transfer, reaction mechanisms

## Abstract

Molecular details for RhoA/GAP catalysis of the hydrolysis of GTP to GDP are poorly understood. We use ^19^F NMR chemical shifts in the MgF_3_^−^ transition state analogue (TSA) complex as a spectroscopic reporter to indicate electron distribution for the γ‐PO_3_^−^ oxygens in the corresponding TS, implying that oxygen coordinated to Mg has the greatest electron density. This was validated by QM calculations giving a picture of the electronic properties of the transition state (TS) for nucleophilic attack of water on the γ‐PO_3_^−^ group based on the structure of a RhoA/GAP‐GDP‐MgF_3_^−^ TSA complex. The TS model displays a network of 20 hydrogen bonds, including the GAP Arg85′ side chain, but neither phosphate torsional strain nor general base catalysis is evident. The nucleophilic water occupies a reactive location different from that in multiple ground state complexes, arising from reorientation of the Gln‐63 carboxamide by Arg85′ to preclude direct hydrogen bonding from water to the target γ‐PO_3_^−^ group.

Hydrolysis of guanosine triphosphate (GTP) by small G proteins (GTPases) of the Ras oncogene superfamily initiates a conformational change that results in On/Off signaling for a wide range of cellular activities.^1^ Slow spontaneous GTP hydrolysis in water (10^−9^ s^−1^) is accelerated by up to 5 orders of magnitude by GTPases, while cooperative GTPase activating proteins (GAPs) further accelerate hydrolysis by 3 to 5 orders of magnitude, raising *k*_cat_ to 5–30 s^−1^.^2^

In structural terms, metal fluoride analogues of the phosphoryl group (PO_3_^−^) have provided new insights into a wide range of phosphoryl transfer reactions. Experimental structures with an octahedral AlF_4_^−^ surrogate for the PO_3_^−^ group were followed by trigonal bipyramidal (tbp) MF_3_ structures,^3^ and later expanded by MgF_3_^−^ as an isosteric and isoelectronic tbp mimic of the PO_3_^−^ group.^4^ These analogues all point to a mechanism for phosphoryl transfer with in‐line geometry in the transition state (TS).1b, 2

In all of the experimental structures of GTPase/GAP TSA complexes, an isolated water is positioned for in‐line attack on Pγ.3b, 5 While general base catalysis (GBC) is regarded as a key catalytic component of phosphoryl transfer enzymes employing a neutral oxygen nucleophile,^6^ there are no structures of GTPases with an amino acid sidechain suitably placed to provide GBC for the attacking water.6a, 7 Substrate‐assisted catalysis, involving direct transfer of a water proton to the O3γ oxygen (atom and RhoA residue numbering is shown in Scheme 1), has been invoked to resolve this problem.^8^ Proton transfer may also be facilitated by a second water in the active site, as supported by computation of model studies in water and their extension to catalysis by Ras/RasGAP.^9^ Time‐resolved Fourier transform IR spectroscopy on a Ras/RasGAP heterodimer, coupled with quantum mechanical (QM) calculations, suggests that non‐bridging oxygens of the α‐ and β‐phosphoryl groups are eclipsed in the ground state.8a, 10

**Scheme 1:**
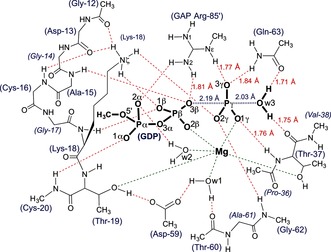
The QM‐derived TS model for GTP hydrolysis by RhoA/RhoGAP showing the H‐bonding network for the catalytic region (red dashes) with ligands coordinated to Mg (green dashes). Amino acid residues are numbered according to RhoA sequence plus Arg85′ from RhoGAP.

^19^F NMR potentially offers a more direct spectroscopic approach to provide a detailed picture of the charge distribution for P−O bond cleavage through TSA complexes mimicking GTP hydrolysis within a small G protein.^11^ Here, we establish the relationship between the RhoA/GAP‐GDP‐MgF_3_^−^ TSA complex (PDB: **1ow3**) and its ^19^F NMR signals to a transition state model for phosphoryl transfer by computational analysis of the structure and electron distributions in both species.

We first made a comparison of 45 GTP, GTP analogue, and GDP‐MF_x_ TSA structures of GTPases to establish i) the degree of commonality in their GTP hydrolysis mechanism, and ii) that RhoA is a typical representative of the superfamily. Using the high resolution (1.65 Å) RhoA/ArhGAP‐GDP‐MgF_3_^−^ TSA complex (PDB: **3msx**) as a standard, protein structures were overlaid with rmsd for Cα alignment in the range 0.2–1.1 Å. They fall into two distinct classes: Michaelis ground state analogue (GSA) complexes and TSA complexes (Figure 1 a; Supporting Information, Table S1). The 32 GSA complexes contain GTP analogues with their isolated nucleophilic water oxygens (Ow3) clustered ≥3.4 Å from Pγ, with an O3β‐P‐Ow3 bond angle deviating significantly from linearity (GPPNP 157±5°). Moreover, Ow3 is close (2.6–3.1 Å) to O3γ of the γ‐PO_3_^−^ group (Figure 1 b), suggesting that the water hydrogen bonds to O3γ. A second hydrogen bond from Ow3 is donated to the C=O of the invariant threonine (Thr37) residue (2.6–3.1 Å). Ow3 also coordinates to the backbone NH group of the invariant Gln residue (Gln63 in RhoA), whose carboxamide sidechain occupies multiple locations (Figure 1 b; Supporting Information, Figure S1).

**Figure 1:**
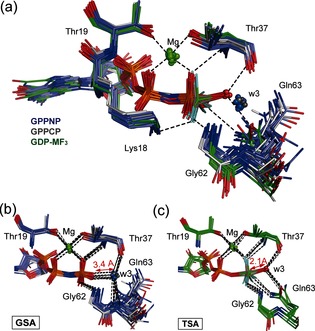
a) Structures of 8 GTPases with GDP bound and MF_3_^−^ ligand in tbp TSA complex (green sticks, in‐line waters in red spheres) are compared with 17 GTPases with GPPNP bound (purple sticks, waters in GS complex positions in blue spheres) and 5 GTPases with GPPCP bound (gray sticks, waters in GS complex positions in gray spheres), all using Cα alignment with PDB: **3msx**. The catalytic Mg (green spheres) and H‐bonds are shown for **1ow3** (black dashes). b) Overlay of 22 GTP analogue structures show coordination for all w3 oxygens (blue spheres) in GS complex positions clustered 3.4 Å from O2γ. c) Overlay of 8 GDP‐MF_3_^−^ structures (Table S1) shows the attacking w3 oxygens (red spheres) coordinated to the central metal at 2.1 Å distance.

By contrast, the 8 tbp MF_3_ structures have attacking waters clustered in‐line with the scissile O−P bond (O3β‐M‐Ow3, 166±6°) and donate hydrogen bonds to both Thr37 and Gln63 C=O sidechain oxygens (Figure 1 c). The fluorine surrogates of the γ‐PO_3_^−^ oxygens are fully coordinated to other residues, with the result that Ow3 is trigonally coordinated with respect to the metal cation (Mg or Al) surrogate of Pγ (2.1±0.1 Å; Table S1). None of these experimental structures have a second water in the active site. These TSA structures display eclipsing of non‐bridge β‐ and γ‐phosphoryl oxygens, arising from chelation to the catalytic Mg (ψ‐dihedral angle −10°), and contrasting with a staggered arrangement of non‐bridge oxygens on the α‐ and β‐phosphoryl groups (ψ‐dihedral angle 64±8°; Table S1).

In solution, ^19^F NMR of the RhoA/GAP‐GDP‐MF_3_^−^ TSA complex gives three clearly resolved resonances that were assigned using solvent‐induced isotope shifts (SIIS; Figure 2). The most shielded fluorine (F_1_, −173.4 ppm; SIIS 0.8 ppm) binds to the catalytic Mg and accepts a single hydrogen bond from the backbone NH of Thr37. The most deshielded fluorine (F_3_, −143.4 ppm; SIIS 1.6 ppm) accepts one hydrogen bond each from Gln63 and the arginine finger (Arg85′) sidechains. The third fluorine (F_2_, −154.3 ppm; SIIS 1.4 ppm) is hydrogen bonded to the NH_3_^+^ group of Lys18 and to the backbone NH of the invariant Switch II Gly residue (Gly62). The observed SIIS values show that none of the fluoride ions are hydrogen bonded to any water molecule in the TSA complex in solution. In addition, the marked upfield shift for F_1_ implies that it has a higher electron density than F_2_ or F_3_.

**Figure 2:**
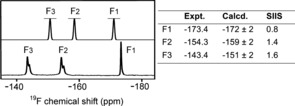
Upper spectrum: Calculated ^19^F NMR for the computational model of the RhoA/GAP‐GDP‐MgF_3_^−^ TSA complex, Lower spectrum: Experimental ^19^F NMR of the RhoA/GAP‐GDP‐MgF_3_^−^ TSA complex. Calculated and experimental ^19^F chemical shifts with the measured SIIS are tabulated with fluorines numbered according to corresponding γ‐oxygens in Scheme 1 (Figure S2).

To validate the geometry of MgF_3_^−^ TSAs as a model of the TS for the phosphoryl transfer step, we performed a DFT calculation on a large active site model to embrace all the hydrogen bonds, implicit in multiple X‐ray structures, that stabilize the TS.^12^ This methodology provides an accurate description of geometry and electronic environment in the TS, and has been used extensively in studies of enzyme catalysis.^13^ We computed a QM model of the TS for RhoA based on the **1ow3** structure by substituting the MgF_3_^−^ core by a trigonal planar PO_3_^−^ moiety with starting P−O bonds 1.71 Å. The optimized geometry of the TS was obtained using the M06‐2X functional and standard TS search methods (Supporting Information).^12^ Importantly, this TS model (Figure 3 c) contained 91 heavy atoms (181 total atoms; Figure 3 b,c), a much larger number than in previous studies (17 atoms;8f 33–37 atoms;^10^ 32 atoms;^14^ 39 atoms8a). The inclusion of the loop atoms for Gly12–Cys16 was essential to obtain a stably protonated state for the sidechain *ɛ*‐NH_3_^+^ group of Lys18, and inclusion of the sidechain of Asp59 achieved the correct orientation of the γ‐PO_3_^−^ with excellent superposition of the computed structure on **1ow3** (Figure 3; Supporting Information, Figure S3).

**Figure 3:**
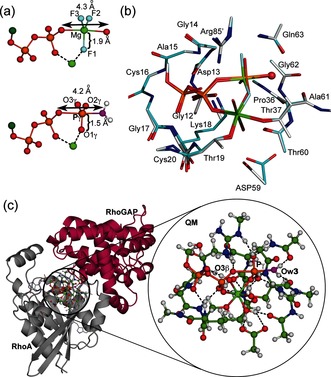
a) Comparison of the geometry of the GDP‐MgF_3_^−^ TSA complex in crystal structure (upper) and the computed PO_3_^−^ TS structure (lower). b) Overlay of atoms in the QM zone in the computed TS (cyan) with **1ow3** (silver). The attacking Ow3 is highlighted as a red sphere. c) Ball‐and‐stick view of the 181 atoms in the QM TS computation. Cartoon representation of QM zone (circled) between RhoA (gray) and RhoGAP (maroon) with the expansion of the QM region in the circle (C, green; H, white; N, blue; O, red; Ow3, magenta; P, orange).

Vibrational frequency analysis of the final model showed that a reliable TS geometry for phosphoryl transfer has been identified (Figure S4). The imaginary mode corresponds to motion of Pγ between the donor and acceptor oxygen atoms along the reaction coordinate (Movie S1). As in **1ow3**, the Gln63 sidechain and the C=O of Thr37 locate the attacking water by accepting its two hydrogen bonds. The equatorial P−O bond lengths about Pγ are 1.51±0.01 Å, typical of the non‐bridging Pγ−O bonds of high‐resolution Mg‐GTP structures (1.52±0.02 Å).^15^ The tbp TS is asymmetric with the Pγ−Ow3 bond length (2.03 Å) slightly shorter than the O3β−Pγ bond length (2.19 Å; Scheme 1). Ow3 remains doubly protonated, even though there is substantial bond formation between Ow3 and Pγ. The ψ‐dihedral angle for the α‐ and β‐phosphoryl groups is staggered (O1α‐Pα‐Pβ‐O1β, 64°; Table S1). Thus, the TS model validates the geometry observed in the high resolution MgF_3_^−^ TSA complex with the expected shortening of the equatorial 1.9 Å Mg−F bonds to the standard 1.5 Å P−O lengths (Figure 3 a,b). In agreement with our deductions based on experimental ^19^F NMR chemical shifts, the oxygen adjacent to the catalytic Mg^2+^ in the γ‐PO_3_^−^ moiety has the highest electron density (Figure 2; Supporting Information).

In a validation study, we replaced the γ‐PO_3_^−^ group in the TS model by trifluoromagnesate and back‐calculated the geometry of a cluster TSA model for the RhoA/GAP‐GDP‐MgF_3_^−^ active site. This new model exhibits almost identical geometry to that seen in **1ow3**, especially about the phosphates, water, and MgF_3_^−^ (rmsd 0.1 Å; Figure S5). The ^19^F NMR chemical shifts computed from this model are in good agreement with experimental results (Figure 2), confirming that the fluorine adjacent to the catalytic Mg^2+^ (F_1_) is the most shielded. This study thus provides independent evidence that O1γ has the largest electron density in the TS for phosphoryl transfer (Figure 3 a). It confirms that ^19^F signals of TSA complexes indeed provide insight into the electronic properties of the corresponding oxygen atoms in the TS for the phosphoryl transfer step, and it implies that O1γ is the strongest base in the proton transfer step. This proposal would be consistent with prior suggestions8c,8d,8g that the Gln63 sidechain plays a role in translocating the proton from w3 to O3γ provided that proton exchange can occur within the γ‐PO_3_^−^ group. However, it is difficult to draw quantitative mechanistic conclusions from QM‐ and QM/MM‐derived active site models in the absence of proper configurational sampling and activation free energy estimates.7c, 9, 16

The close agreement between our spectroscopic, structural, and computational studies enabled a rigorous analysis of the atomic details of small G protein complexes. The clear distinction between those involving unreactive GTP analogues and TSA complexes (Figure 1 and Figure S1) is manifest in critical differences in hydrogen bonds between protein, nucleotide, and isolated water. In the GPPNP and GPPCP complexes, the isolated water is unproductively hydrogen bonded to oxygen O3γ of the γ‐PO_3_^−^ group as well as to the Thr37 C=O group. As a result, the lone pair electrons of Ow3 are directed away from Pγ, preventing nucleophilic attack on the γ‐PO_3_^−^ group. Moreover, in many cases, Ow3 is hydrogen bonded to a nearby N−H group, further reducing its nucleophilicity both geometrically and electrostatically (Figure 1 b, Scheme 2; Supporting Information, Figure S1 and Table S1). The presence of an NH or CH_2_ substitute for the β,γ‐bridging oxygen in GTP usually leads to conformational reorganization of Gln63 into a catalytically inactive position.

**Scheme 2:**
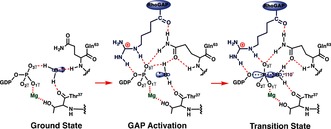
Proposed hydrogen bond coordination (red dashes) to the inert isolated water (w3) in the GSA complex (left); this is converted into an GAP activated conformer (center) by insertion of arginine finger (blue), which permits access to the transition state (right).

The behavior of four GTP complexes of small G proteins that have been crystallized without hydrolysis of GTP (PDBs: **1z0j**, **2c5l**, **1n6l,** and **1wa5**) can now be readily rationalized. In each case, the attacking water donates hydrogen bonds to O3γ (2.9±0.1 Å) and to the Thr37 C=O (2.9±0.1 Å), and is close to the backbone NH of Gln(Leu)63 (3.2±0.2 Å). The water oxygen is at van der Waals distance from Pγ (3.4±0.1 Å) and the O‐P‐O angle is off‐line, similar to that seen for GSA complexes (Table S1 and Figure S6). In three of these complexes, the water has planar trigonal coordination to its three heavy atom neighbors (improper angle 9.0±0.2°), while in the fourth it is tetrahedrally coordinated. Therefore, the water lone pair electrons cannot interact with an antibonding σ* state for the scissile O3β−Pγ bond in any of these four complexes (Scheme 2; Supporting Information, Figure S6). These complexes are thus rendered passive by the isolated water donating a hydrogen bond to the target γ‐phosphoryl group (Figure 1 b). It follows that in‐line geometry is a necessary, but not a sufficient, criterion for approach to the TS for phosphoryl group transfer. Moreover, it seems probable that the stability of GTP (and of ATP) to spontaneous hydrolysis in bulk water results from multiple hydrogen bonding of water molecules to all 7 phosphoryl oxygens, with consequent disruption of in‐line nucleophilic attack.

The rate acceleration provided by GAPs has been broadly attributed to the insertion of an arginine finger into the active site,^17^ where it provides electrostatic catalysis,7c, 8f makes the β‐phosphate a better leaving group by hydrogen bonding to the β,γ‐bridging oxygen (O3β), and excludes solvent.^17^, ^18^ Our results identify two additional roles for Arg85′. First, it donates a hydrogen bond to O3γ and thus helps rupture the deactivating hydrogen bond between O3γ and w3. Second, the hydrogen bond between Arg85′ C=O and Gln63−NH_2_ orientates the latter to hydrogen bond to O3γ. The Gln63 carboxamide oxygen thereby accepts a hydrogen bond from w3, which remains hydrogen bonded to the Thr37 C=O and is aligned for nucleophilic attack on Pγ with the required orientation (computed O‐P‐O angle 177° compared with 156° for GSA structures) and distance (Pγ−Ow3 2.04 Å; Figure 3 a). The progression from hydrogen bonded GS complex to the GAP‐activated complex, and thence to the TS, is seen to depend on protein control of hydrogen bonds to the isolated water and to the transferring PO_3_^−^ group (Scheme 2).

In conclusion, the strong contrast between the GDP‐MgF_3_^−^ TSA structures and the broad range of GTP analogue structures establishes that MgF_3_^−^ is a prime mimic of the TS that is formed following conformational switching of key functional residues into their catalytically active positions. With MgF_3_^−^ TSAs, ^19^F NMR is a simple approach to evaluate contributions made by specific residues to catalytic function and TS stabilization, since the ^19^F chemical shifts accurately reflect electronic properties relating to the oxygens of the transferring phosphoryl group.^11^ Moreover, the sensitivity of ^19^F chemical shifts in response to surrounding charge distribution, and to hydrogen bonding partners in solution, signal effects that are inaccessible to other structural methods.

## Supporting information

As a service to our authors and readers, this journal provides supporting information supplied by the authors. Such materials are peer reviewed and may be re‐organized for online delivery, but are not copy‐edited or typeset. Technical support issues arising from supporting information (other than missing files) should be addressed to the authors.

Supplementary

Supplementary
