# Supplementary material for: 19F NMR and DFT Analysis Reveal Structural and Electronic Transition State Features for RhoA‐Catalyzed GTP Hydrolysis
Source: Angew Chem Int Ed Engl. 2016 Jan 28;55(10):3318–22. doi: 10.1002/anie.201509477 (PMC4770445; doi:10.1002/anie.201509477)
Supplement: Supplementary file 1 — Supplementary [file ANIE-55-3318-s001.pdf]

Angewandte Chemie
Eine Zeitschrift der Gesellschaft Deutscher Chemiker

Supporting Information

^{19}F NMR and DFT Analysis Reveal Structural and Electronic Transition State Features for RhoA-Catalyzed GTP Hydrolysis

Yi Jin+, Robert W. Molt, Jr.+, Jonathan P. Waltho, Nigel G. J. Richards,* and G. Michael Blackburn**

anie_201509477_sm_miscellaneous_information.pdf
anie_201509477_sm_nucleophilic_attack.mp4

Table of Contents

1. Comparison of small G protein structures.
2. Gene Expression and protein purification for RhoA and RhoGAP
3. ^{19}F NMR investigation of a TSA complex.
4. Obtaining the TS model.
5. Obtaining the calculated active site model for the RhoA/GAP-GDP-MgF_3^- complex.
6. NMR chemical shift calculations.
7. Calibrating the error associated with the ^{19}F NMR chemical shift calculations.
8. Dissociative/associative mechanism.
9. **Movie S1.** Visualization of the characteristic imaginary vibrational mode of the TS model in which the water nucleophile attacks the γ-phosphoryl group of GTP.
10. **Table S1.** Structural data for 45 substrate, inhibitor, and tbp TSA complexes of small GTPases.
11. **Table S2**. Selected internal coordinate values for the computational TS model, the RhoA/GAP-GDP-MgF_3^- X-ray crystal structure (**1ow3**) and the calculated active site model for the RhoA/RhoGAP-GDP-MgF_3^- complex.
12. **Figure S1**. Michaelis complex structures for Ras superfamily GTPases with GTP analogs.
13. **Figure S2**. **Experimental ^{19}F NMR of the RhoA/GAP-GDP-MgF_3^- TSA complex in 100% H_2O and 100% D_2O.**
14. **Figure S3**. Inclusion of Asp59 in the model is important for the correct computation of transition state.
15. **Figure S4**. The final computed TS structure for the RhoA/GAP-GTP complex.
16. **Figure S5**. Computed MgF_3^- TSA structure for RhoA/GAP-GDP-MgF_3^- complex.
17. **Figure S6**. Cross-eyed stereo view showing H-bonded Michaelis complex structures for 4 Ras superfamily GTPases with GTP.
18. **References**

Comparison of small G protein structures. In order to establish the extent of commonality in the mechanism of GTP hydrolysis by small G proteins, and also to ascertain whether RhoA is a suitable representative of the superfamily, a comparison of 45 deposited GTP, GTP analogue, and GDP-MF_x TSA structures of GTPases was undertaken, with data shown in Table S1. They comprise 4 GTP□structures (entries 1-4), 17 GPPNP structures (entries 5-21), 6 GPPCP structures (entries 22-27), 9 GTPγS structures (entries 28-37)[†], and 8 TSA structures with GDP-MF_3 as tbp TSA (entries 38-45). Data are taken from the respective pdb files using PyMol.[1] Using the high resolution (1.65 Å) RhoA/ArhGAP-GDP-MgF_3^- TSA complex (PDB: **3msx**) as the archetype, the protein structures overlaid with rmsd for C_α alignment in the range 0.2 - 1.1 Å. The structures fall into two distinct classes: Michaelis complexes and transition state analogs (Figure 1a, Table S1).

The 32 GTP analogue complexes have the isolated water (w3) more than 3.4 Å from Pγ and the O3β-Pγ-Ow3 bond angle deviates significantly from linearity (157˚ ± 5˚ for GPPNP, 155˚ ± 6˚ for GPPCP, and 161˚ ± 4˚ for GTPγS structures). This indicates that nucleophilic attack requires further structural rearrangement. Moreover, in these complexes there is a well-defined H-bond (2.6 - 3.1 Å heavy atom separation) donated by the attacking water to oxygen O3γ of the γ-PO_3^- group (Figure 1). The second H-bond donated from this water is to the carbonyl oxygen of the invariant threonine residue (Thr37 in RhoA) (2.6 – 3.1 Å) (Table S1). This water is also typically coordinated by the backbone amide NH group of the invariant glutamine residue (Gln63 in RhoA), while the side-chain of this glutamine occupies multiple conformations, many displaced from this water molecule (Figure 1b and Figure S1).

By contrast, in the 8 trigonal bipyramidal (tbp) MF_3 TSA structures, the isolated water is not H-bonded to the γ-PO_3^- group but always donates H-bonds to the carbonyl oxygens of *both* Thr37 *and* the Gln63 side-chain (Figure 1c). As a result, this water oxygen is now trigonally coordinated with respect to the proximate heavy atoms, including the metal surrogate of Pγ (2.1 ± 0.1 Å), and is more closely aligned with the breaking O-Pγ bond (O3β-M-Ow3 bond angle 166˚ ± 6˚) (Table S1). All 8 TSA structures show significant eclipsing of the non-bridging oxygens on the β- and γ-phosphoryl groups arising from their chelation to the catalytic Mg (ψ-dihedral angle -10˚), but staggering of the oxygens on the α- and β-phosphoryl groups (ψ-dihedral angle 64˚ ± 8˚) (Table S1). Only one outlier structure, the Ras/RasGAP-GDP-MF_3^- TSA complex (**1wq1**),[2] shows atypical structural parameters, with an O1β-Pβ-Pγ-O2γ ψ-dihedral angle of -2.5˚ and an O1α-Pα-Pβ-O1β ψ-dihedral angle of 46˚.

Gene Expression and protein purification for RhoA and RhoGAP. Plasmids expressing N-terminal GST-tagged RhoGAP (fragment 198–439) and N-terminal GST-tagged GST-$RhoA_{F25N}$ were generously given by Dr. K. Rittinger (MRC National Institute for Medical Research, London). Both proteins were expressed in *E. coli.* BL21-Rosetta-pLysS or Rosetta 2 strains in LB media. Expression was induced with 1 mM IPTG for 6 h at 30 °C or overnight at 20 °C, at 250 rpm. Both RhoA and RhoGAP were purified following the same protocol. After cells were lysed by sonication in Lysis Buffer (50 mM Tris, pH 7.6, 50 mM NaCl, 5 mM $MgCl_2$, 1 mM DTT), the clear lysate was loaded on glutathione agarose column and incubated at 4 °C on a rotating wheel for 1 h before being washed with thrombin digestion buffer (50 mM Tris, pH 8.0, 150 mM NaCl, 5 mM $MgCl_2$, 1 mM DTT). The on-column thrombin digestion was carried out on beads overnight at 4 °C on a rotating wheel and the flow-through containing impure RhoGAP or RhoA was collected. The protein was further purified

---

[†] Abbreviations used: Abbreviations: GDP, guanosine 5'-diphosphate; GTP, guanosine 5'-triphosphate; GPPNP, β,γ□□□□□□□□□□□□□□□□□□□β,γ□□□□□□□□□□□□□□□□□□□□γ□□□γ□□□□□□□ □□□

on a pre-equilibrated S75 Superdax Gel filtration column (50 mM Tris, pH 8.0, NaCl mM 150, 5 mM $MgCl_2$, 1mM DTT).

^{19}F NMR investigation of a TSA complex. The RhoA/GAP complex was shown previously to be amenable to solution NMR,[3] and crystallization,[4] including two of the best-resolved trifluoromagnesate TSA complex structures of small G proteins (**1ow3**, 1.80 Å and **3msx**, 1.65 Å). All NMR experiments used a D_2O capillary as deuterium lock except experiments for isotope shift measurement, done in 100% D_2O. 1D ^{19}F NMR spectra were recorded on a Bruker Avance 500 MHz spectrometer equipped with a 5 mm dual $^{1}H/^{19}F$ probe. Typically, 32k scans were acquired over a spectral width of 200 ppm with carrier frequency set to –140 ppm. In order to suppress the free MgF^{+} resonance (-155 ppm) by saturation transfer, continuous-wave ^{19}F radiation for the ^{19}F NMR experiments of the RhoA/GAP-GDP-MgF_3^- TSA complex was applied to the free fluoride resonance (-120 ppm) with a power level of 42 dB over a 1 s recycle delay. This completely removed signal overlap between the broad MgF^{+} resonance and the F_2 resonance. Samples of RhoA/GAP-GDP-MgF_3^- TSA complex contained 1.0 mM RhoA (GDP 1:1 bound), 1.1 mM RhoGAP in 5 mM Tris buffer, pH 7.4, with 150 mM NaCl, 10 mM $MgCl_2$, 10 mM NH_4F, and 1 mM deferoxamine. In contrast to the RhoA/GAP-GDP-AlF_4^- TSA complex, which showed one rotationally averaged signal,[3] the ^{19}F NMR resonances for the individual fluorines in the trifluoromagnesate complex were clearly resolved (Figure 2). This illustrates the improved ability of the enzyme to restrict the rotation of the equatorial atoms when binding a tbp rather than an octahedral TSA mimic.[5] The ^{19}F resonances span ~30 ppm and were readily assigned on the basis of solvent-isotope induced shifts (SIIS) by comparing the chemical shift differences in 100 % H_2O and 100 % D_2O buffers (Figure 2). SIIS values accurately reflect the distance of solvent-exchangeable hydrogens from the fluorine nuclei in metal fluoride complexes.[5-6] The most shielded fluorine (F_1, –173.4 ppm; SIIS 0.8 ppm) binds to the catalytic Mg and accepts a single H-bond from the backbone NH of Thr37. The most deshielded fluorine (F_3, –143.4 ppm; SIIS 1.6 ppm) accepts one H-bond each from Gln63 and the arginine finger (Arg85') side-chains. The third fluorine (F_2, –154.3 ppm; SIIS 1.4 ppm) is H-bonded to the NH_3^+ group of Lys18 and the backbone NH of the invariant Switch II Gly residue (Gly62).

The ^{19}F spectra of the RhoA/GAP-GDP-MgF_3^- TSA complex reveal a minor population of a second conformer (Figure S2). No resolved second resonance is observed for F_3, and the changes in chemical shift for F_2 (0.7 ppm) and F_3 (0.6 ppm) are too small for a change of the number of H-bonds in which they are involved. This conclusion is supported by the lack of any measureable changes in the SIIS values for these resonances between the major and minor forms. The direction of chemical shift changes is consistent with the H-bonding partners being slightly more distant from F_2 and F_3 in the minor form, but not changed sufficiently to alter the SIIS values measurably. A likely rationale is that a conformational change peripheral to the active site is causing the Switch II region (including G62 and Q63) not to pack quite as tightly to the MgF_3^- moiety in the minor form. There is no clear evidence in the crystal structures for the source of this change, and its true identity requires further investigation.

Obtaining the TS model. Our model for the transition state (TS) of the γ-phosphate hydrolysis reaction was obtained using Kohn-Sham Density Functional Theory (KS-DFT). We used the M06-2X functional formulation of KS-DFT.[7] A cc-pVDZ basis set was used to represent single-particle wavefunctions for all atoms excepting atoms for which more care was given due to reaction importance. Many atoms were expected to have a higher negative charge density (for example, the oxygens along GTP) and therefore require diffuse functions. Additionally, all of the atoms directly involved in the bond formation/breaking also had a triple zeta basis set given that these atoms are more important. Thus, the nucleophilic water oxygen had aug-cc-pVTZ (oxygen requiring diffuse

functions due to possible charge density buildup) and the hydrogens had cc-pVTZ (hydrogens never require diffuse functions). The oxygens of the phosphoryl group had aug-cc-pVTZ given their higher charge density, whereas the phosphorus had cc-pVTZ (since it is more positive charged and therefore less in need of diffuse functions). The 03β oxygen of GDP was given the aug-cc-pVTZ basis set for similar rationales. As the remaining oxygens of GDP are of secondary importance, these oxygens received aug-cc-pVDZ basis functions (which proved essential to get the highly sensitive NMR correct). All phosphorus and magnesium atoms used cc-pVTZ given that they have greater polarizability and should therefore have greater basis flexibility to reflect that polarizability. Fluorines utilized aug-cc-pVTZ in the magnesium trifluoride for the same rationale as oxygens in the phosphoryl group [8]. The active site (cluster) model (Figure 3c) was constructed so as to maintain all key hydrogen bonding capable of stabilizing the transition state. The initial geometry about the γ-phosphorus atom was obtained by replacing the tbp magnesium by phosphorus and the three fluorines by oxygens in the high-resolution X-ray structure (**1ow3**). More specifically, we included atoms in residues 12-19, 36-38, and 59-63 (from RhoA) and 85' (from RhoAGAP). All amino acid hydrogen bonds stabilizing the attacking water, the γ-phosphoryl group, or leaving group were included. Waters bonded to the catalytic magnesium were also retained. Where opportune, we truncated amino acid residues with a methyl group in which the carbon was fixed at the crystallographic coordinates of the cognate atom in the X-ray crystal structure. Initially, the TS search utilized cc-pVDZ for all atoms and an integration grid consisting of 99 radial points and 590 solid-angle points in the Lebedev grid. Upon calculating an initial TS structure in this manner, we increased the quality of the calculation for greater accuracy and to eliminate spurious small imaginary frequencies. We added basis functions in the manner most conducive of better representing the polarizations of different atoms (see above). The final refined integration grid had 160 radial points, 974 solid angle points. The structure was considered optimized when the force on all nuclei fell below 1 μHartree/Bohr. The SCF was considered converged when the density matrix residual was less than 10^{-6}. After decreasing the initial 1.91 Å Mg-F distance to a value of 1.71 Å for the three new P-O bonds, we optimized the geometry of the resulting active site model (181 atoms) to obtain the TS using standard algorithms,[9] as implemented in the Gaussian09 software package.[10] All "terminal methyl" carbons were fixed at their initial locations in **1ow3**, which did not introduce any significant error into the calculation. This procedure gave a converged TS model with a harmonic vibrational value of 191*i* cm^{-1} corresponding to motion along the reaction coordinate (see movie S1). However, in freezing the Cartesian coordinates associated with the terminal methyl groups, there were a small number of non-relevant imaginary frequencies associated methyl group librations (31*i* cm^{-1}, 25*i* cm^{-1}, 18*i* cm^{-1}, 11*i* cm^{-1}, 9*i* cm^{-1}). Coordinates for the TS model are available on request (RichardsN14@cardiff.ac.uk). By partitioning the total electron density into localized orbitals, cognisant of the approximations inherent to local orbital representation, analysis of the three equatorial P–O bonds in the computed TS identified them as each having 33% s character, 65% p character, and 2% d character. These three oxygens can generally be described as having sp^3 hybridisation, which suggests that the equatorial P-O bonds are close to simple sp^2 hybrids with virtually no pπ-dπ double-bond character. This was obtained using standard NBO analysis.

Obtaining the calculated active site model for the RhoA/GAP-GDP-MgF_3^- complex. An active site model for the RhoA/GAP-GDP-MgF_3^- complex was obtained from the atomic coordinates of the TS model except that the P and O atoms in the γ-phosphoryl group were replaced by Mg and F, respectively. The optimized structure was obtained using a similar computational protocol to that used for the TS model except that standard optimization algorithms were used to find the ground state structure. Given the electronegativity of fluorine, it was necessary to add diffuse functions in the

form of an aug-cc-pVTZ basis on the fluorine atoms.[11] Coordinates for the RhoA/GAP-GDP-MgF_3^- active site model are available on request (RichardsN14@cardiff.ac.uk).

NMR chemical shift calculations. NMR shielding tensors for ^{17}O and ^{19}F nuclei in the TS model and the calculated RhoA/GAP-GDP-MgF_3^- complex active site model, respectively, were computed from the coupled-perturbed Hartree-Fock equation and gauge-invariant atomic orbitals derived from the DFT electron densities using standard algorithms implemented in the Gaussian09 software package.[10, 12] The values of the shielding tensor for the fluorines are 327.4, 312.2, and 305.3 for F1, F2, and F3 respectively using aug-cc-pVTZ for the fluorines. When the basis is increased to aug-cc-pVQZ, the values become 325.6, 310.8, and 304.0 respectively, demonstrating that we are quite converged. The same relative ordering in most shielded nucleic exists for oxygens as well as fluorines. For O1, O2, and O3, the aug-cc-pVDZ shieldings are 168.4, 150.4, and 147.2, mirroring the same trends as fluorines. When we increase the basis to aug-cc-pVTZ, the values are 154.5, 135.0, and 130.8. We adapted the expression of our absolute shielding tensors into solvent-adjusted chemical shifts via a database of solvent fluorine NMR[14] using established methods of relating gas-phase NMR shielding tensors to solution-phase chemical shifts.[15] We remain open-minded that statements of the most shielded atomic nucleus is not a 1-1 mathematical statement of the reaction mechanism, as a higher electron density before proton transfer is at best a kinetic statement, rather than a thermodynamic statement.

Dissociative/Associative Mechanism. Our model has a direct bearing on the question of associative versus dissociative nature of the transition states, which remains controversial, particularly in computational studies. There is little deviation from planarity of the γ-PO_3^- moiety, the Arg85’ finger of RhoGAP interacts with oxygen atoms on each phosphoryl group, and the O3β-Pγ-Ow3 angle (175°) is consistent with in-line attack. The geometry of our TS model addresses whether phosphoryl transfer in RhoA/GAP proceeds *via* an associative or a dissociative TS.[16] Early analyses of the TS for phosphoryl transfer focused on a boundary of 4.9 Å for the separation of the apical oxygen atoms in fully dissociative processes.[17] For our TS model this O--O distance is 4.26 Å and, by the above definition, is consistent with an associative contribution to the concerted TS, in line with other mechanistic proposals.[18] Intriguingly, the axial O--O distance for the 8 tbp MF3 TSA analogs (4.27 ± 0.13 Å) is well within error of the value for the computed TS model (Figure 3b). The relative P--O distances for the departing oxygen (2.19 Å) and incoming oxygen (2.03 Å) indicate that bond breaking is hardly more advanced than bond making for the hydrolysis of GTP (Scheme 1), suggestive of partial associative character within a compact, concerted ANDN transition state. Our TS model contrasts with the proposal of a fully dissociative DN + AN mechanism based on a previous computation built from a Ras TSA structure (**1wq1**) and a Ras-GTP structure lacking a GAP protein (1qra),[19] a discrepancy that highlights the problem of modeling phosphoryl transfer with an inadequate number of QM atoms.[20] It seems likely that the transition states for Cdc42, Rab, Rac, and Ras will possess all the key structural features observed here for RhoA.

Movie S1. Visualization of the characteristic imaginary vibrational mode of the TS model in which the water nucleophile attacks the γ-phosphoryl group of GTP. Images were created in GaussView V5.0[21] and are looped for ease of viewing. The scalar amplitude of the atomic motions along the eigenvector associated with this vibrational mode is defined by a factor A, which is computed according to the following equation:

$$A = 0.1 \times [2.0 + (S - 2.0) \times (13.0 / 98.0)]$$

where S is an integer value that ranges from 2 to 100. Hence, A ranges from 0.2-1.5 Å.

Table S1. Structural data for 45 substrate, inhibitor, and tbp TSA complexes of small GTPases.

Entry	Ligand	PDB Entry	Structure Resolution	Ow3-Pγ	Ow3-O3γ	Ow3-T37	Ow3-NH	O-Pγ-O angle	O-Pγ-O distance	Wat3 imp-dihedral	O1α-Pα-Pβ-O1β ψ-dihedral	O1β-Pβ-Pγ-O3γ ψ-dihedral
1	GTP	**1z0j**	1.32 Å	3.34 Å	3.05 Å	2.76 Å	3.07 Å (L63NH)	167.1°	4.95 Å	8.9°	74.28°	4.95°
2	GTP	**1n6l**	1.60 Å	3.55 Å	2.87 Å	3.05 Å	2.94 (Q63 NH2)<br>3.38 (Q63NH)	156.4°	5.04 Å	*48.14°*<br>*ψ-tetrahedral*	66.83°	-7.16°
3	GTP	**2c5l**	1.90 Å	3.26 Å	2.94 Å	2.82 Å	3.07 Å (Q63NH)	163.2°	5.10 Å	9.17°	70.52°	-15.21°
4	GTP	**1wa5**	2.00 Å	3.37 Å	2.71 Å	2.86 Å	3.22 Å (Q69NH)	161.12°	4.93 Å	8.93°	61.57°	25.70°
	**Mean and SD**			3.38 ±0.12 Å	2.89 ± 0.14 Å	2.87 ± 0.13 Å	3.2 ± 0.15 Å	162° ± 4.5°	5.00 ± 0.08 Å	9.0° ± 0.15° [for three]	68.3° ± 5.4°	2° ± 18°
5	GNP	**3x1z**	1.25 Å	3.48 Å	2.88 Å	2.85 Å	3.36 Å (T61)	161.17°	5.08 Å	15.79°	73.76°	-8.07°
6	GNP	**3tgp**	1.31 Å	3.43 Å	2.74 Å	2.76 Å	2.83 Å (Q61)	159.93°	4.97 Å	*44.9°*	72.4°	-6.69°
7	GNP	**5p21**	1.35 Å	3.69 Å	2.85 Å	2.97 Å	3.50 Å (G60)	157.59°	5.24 Å	25.4°	71.68°	-11.49°
8	GNP	**3i3s**	1.36 Å	3.43 Å	2.82 Å	2.89 Å	3.09 Å (Q61)	162.58°	5.04 Å	12.57°	67.75°	-10.57°
9	GNP	**1n6h**	1.51 Å	3.62 Å	2.88 Å	3.05 Å (Q61 C=O)	3.11 Å (Q61)	149.93°	5.08 Å	*53.97°*	72.98°	-15.99°
10	GNP	**1kmq**	1.55 Å	3.44 Å	2.98 Å	2.80 Å	3.08 Å	163.91°	5.01 Å	18.9°	64.84°	-11.91°
11	GNP	**4hb2**	1.80 Å	3.49 Å	2.69 Å	3.00 Å	3.11 Å (Q69)	152.75°	5.08 Å	11.4°	66.46°	-19.16°
12	GNP	**1huq**	1.80 Å	3.72 Å	3.08 Å	2.80 Å	2.84 Å (Q61NH2)	158.37°	6.71 Å	*42.99°*	73.57°	-20.01°
13	GNP	**1n6o**	1.80 Å	3.57 Å	2.76 Å	3.08 Å	2.90 Å (Q79NH2)	151.04°	5.15 Å	*46.41°*	70.64°	-17.06°
14	GNP	**4js0**	1.90 Å	3.46 Å	2.80 Å	2.94 Å	3.47 Å	157.20°	5.01 Å	-19.57°	65.36°	-7.56°
15	GNP	**4l9w**	1.95 Å	3.39 Å	2.74 Å	3.03 Å	3.16 Å (Q61NH)	158.07°	4.94 Å	-16.96°	65.70°	-10.32°
16	GNP	**3rab**	2.00 Å	3.51 Å	3.11 Å	3.28 Å	3.27 Å (Q81NH)	164.42°	5.12 Å	-10.59°	63.66°	*-24.72°*

							3.34 Å (Q87NH_2)					
17	GNP	**4bas**	2.00 Å	3.48 Å	2.67 Å	2.88 Å	3.42 Å (A63NH)	148.35˚	5.03 Å	-19.50˚	63.54˚	-10.48˚
18	GNP	**3qbt**	2.00 Å	3.64 Å	2.93 Å	2.84 Å	3.17 Å (Q67NH)	156.11˚	5.22 Å	-6.75˚	66.74˚	-13.86˚
19	GNP	**1g17**	2.00 Å	3.44 Å	2.87 Å	2.97 Å	3.02 Å (Q79NH)	164.10˚	5.02 Å	-14.19˚	64.66˚	-18.26˚
20	GNP	**1nf3**	2.10 Å	3.31 Å	2.70 Å	2.98 Å	3.27 Å (L61)	160.25˚	4.93 Å	-16.26˚	56.10˚	-5.12˚
21	GNP	**4m9q**	2.50 Å	3.62 Å	2.66 Å	2.77 Å	2.96 Å	151.42˚	5.18 Å	-16.64	62.23˚	-19.25˚
	**Mean and SD**			3.51 ± 0.11 Å	2.81 ± 0.19 Å	2.93 ± 0.13 Å	3.15 ± 0.21 Å	157.5˚ ± 5.2˚	5.17 ± 0.41 Å	23.1˚ ± 14.5˚ 15.7˚ ± 4.8˚	68.0˚ ± 5.4˚	-13.6˚ ± 5.5˚ -12.9˚ ± 4.9˚
23	GCP	**121p**	1.54 Å	3.56 Å	2.64 Å	2.84 Å	3.13 Å (Wat)	156.41˚	5.16 Å	-11.9˚	79.71˚	-10.3˚
24	GCP	**2qme**	1.75 Å	3.66 Å	2.94 Å	2.87 Å	2.82 Å (Q61NH_2)	154.84˚	5.36 Å	-46.82˚	70.59˚	-13.31˚
25	GCP	**6q21**	1.95 Å	3.30 Å	2.64 Å	2.88 Å	2.70 Å (Q61NH_2) 2.85 Å (Q61NH)	165.04˚	5.85 Å	-43.77˚ -11.66˚	95.63˚	-23.22˚
26	GCP	**4dsn**	2.03 Å	3.33 Å	2.67 Å	2.81 Å	3.15 Å (Wat)	155.81˚	5.04 Å	-5.62˚	73.97˚	0.70˚
27	GCP	**2ov2**	2.10 Å	3.48 Å	2.74 Å	2.66 Å	2.92 Å (Q61NH_2) 3.06 Å (Q61NH)	150.98˚	5.14 Å	-9.53˚	77.19˚	-12.84˚
28	GCP	**4dst**	2.30 Å	3.31 Å	2.76 Å	2.63 Å	3.03 Å (G60)	147.36 ˚	5.03 Å	-23.96˚	70.16˚	2.52˚
	**Mean and SD**			3.44 ± 0.15 Å	2.73 ± 0.11 Å	2.78 ± 0.11 Å	2.96 ± 0.18 Å	155˚ ± 6˚	5.26 ± 0.31 Å	-22˚ ± 17˚	77.9˚ ± 9.5˚	-10.5˚ ± 8.2˚
29	GSP	**2ffq**	1.78 Å	3.94 Å	3.26 Å	2.51 Å	2.99 Å (Q72NH_2)	155.48˚	5.47 Å	30.00˚	70.57˚	-20.14˚
30	GSP	**3reg**	1.80 Å	3.92 Å	3.25 Å	2.95 Å	2.85 Å (Q78?)	158.88˚	5.46 Å	29.13˚	63.86˚	-25.42˚
31	GSP	**4ds0**	1.85 Å	3.62 Å	3.11 Å	2.65 Å	3.56 Å (Q61NH_2) 3.75 Å (Q61NH)	162.38˚	5.23 Å	22.98˚ -11.21˚	68.89˚	-20.55˚
32	GSP	**1aso**	2.00 Å	3.83 Å	3.19 Å	2.73 Å	2.95 Å (Q204NH_2)	156.17˚	5.38 Å	31.43˚	73.66˚	11.30˚
33	GSP	**1gia**	2.00 Å	3.85 Å	3.27 Å	3.00Å	3.75 Å (Q205NH)	161.33˚	5.44 Å	15.66˚	67.05˚	-15.80˚
34	GSP	**2gcp**	2.15 Å	3.67 Å	3.18 Å	2.64 Å	2.94 Å (Q63?) 3.44 Å (Q63NH)	165/75˚	5.29 Å	28.67˚	58.74˚	-19.53˚
35	GSP	**1cxz**	2.20 Å	3.75 Å	3.31 Å	2.72 Å	3.23 Å (Q63NH)	167.82˚	5.39 Å	-14.97˚	64.98˚	-24.55˚

36	GSP	**2fju**	2.20 Å	3.67 Å	3.19 Å	2.70 Å	3.19 Å (Q61?)	160.44˚	5.25 Å	28.73˚	68.19˚	-13.06˚
37	GSP	**2w2x**	2.30 Å	3.88 Å	3.23 Å	2.64 Å	3.49 Å (Q61NH)	164.81˚	5.44 Å	-11.39˚	80.53˚	-31.54˚
	**Mean and SD**			3.79 ± 0.12 Å	3.22 ± 0.06 Å	2.73 ± 0.16 Å	3.21 ± 0.32 Å	161.5˚ ± 4.2˚	5.37 ± 0.09 Å	22.4˚ ± 8.7˚	68.5˚ ± 5.2˚	20.2˚ ± 6.4˚
38	GDP.AlF_3	**1n6k**	1.55 Å	2.14 Å	-	3.06 Å	2.68 Å	164.73˚	4.09 Å	-7.01˚	65.78˚	-23.21˚
39	GDP.MgF_3	**3msx**	1.65 Å	2.11 Å	-	2.75 Å	2.81 Å (Q63CO)	169.13˚	4.19 Å	-5.88˚	57.48˚	-7.61˚
40	GDP.MgF_3	**1ow3**	1.80 Å	2.11 Å	-	2.89 Å	2.73 Å (Q63CO)	172.38˚	4.19 Å	-4.78˚	61.62˚	5.07˚
41	GDP.AlF_3	**2ngr**	1.90 Å	2.11 Å	-	2.85 Å	3.09 Å (Q61CO)	168.17˚	4.22 Å	6.89˚	61.98˚	-12.69˚
42	GDP.AlF_3	**1he1**	2.00 Å	2.23 Å	-	2.71 Å	2.68 Å (Q61CO)	171.70˚	4.25 Å	0.72˚	62.82˚	-5.65˚
43	GDP.AlF_3	**1grn**	2.10 Å	2.26 Å	-	2.69 Å	2.80 Å (Q61CO)	157.49˚	4.39 Å	17.18˚	54.81˚	-10.03˚
44	GDP.AlF_3	**1wq1**	2.50 Å	2.20 Å	-	3.03 Å	2.93 Å (Q61CO)	165.13˚	4.45 Å	14.04˚	*45.96˚*	-2.49˚
45	GDP.AlF_3	**4iru**	3.20 Å	1.99 Å	-	2.72 Å	2.75 Å (Q70CO)	158.00˚	4.39 Å	-6.93˚	79.48˚	-11.09˚
	**Mean and SD**			2.14 ± 0.09 Å	-	2.84 ± 0.15 Å	2.81 ± 0.14 Å	165.8˚ ± 5.9˚	4.27 ± 0.13 Å	7.9˚ ± 5.2˚	63.4˚ ± 7.9˚	-9.7˚ ± 6.4˚
46	GDP.MgF_3	computed		2.04 Å.	-	2.84 Å	2.70 Å (G63CO)	177.17	4.06 Å	-2.30˚	66.66˚	-0.92˚
40	GDP.MgF_3	**1ow3**	1.80 Å	2.11 Å	-	2.89 Å	2.73 Å (Q63CO)	172.38˚	4.19 Å	-4.78˚	61.62˚	5.07˚
47	GTP	computed		2.03 Å	-	2.76 Å	2.70 Å (Q63CO)	173.49˚	4.22 Å	-14.67˚	65.59˚	-9.72˚

Table S2. Selected internal coordinate values for the computational TS model, the RhoA/GAP-GDP-MgF_3^- X-ray crystal structure (1ow3) and the calculated active site model for the RhoA/RhoGAP-GDP-MgF_3^- complex. Atom names correspond to those shown in Figure 2 of the main text. For the calculated structures, bond lengths, bond angles and dihedral angles are not more accurate than 0.02Å, 1° or a few degrees, respectively.

**Internal Coordinate**	**TS Model (PO_3^-)**	**1ow3 (MgF_3^-)**	**TSA model (MgF_3^-)**
**Bond Lengths (Å)**			
P_γ-O(w3)	2.03	n.a	n.a
P_γ-O1γ	1.52	n.a	n.a
P_γ-O2γ	1.50	n.a	n.a
P_γ-O3γ	1.51	n.a	n.a
P_γ-O3β	2.19	n.a	n.a
Mg-O(w3)	n.a.	2.11	2.04
Mg-F_1	n.a.	1.85	1.91
Mg-F_2	n.a.	1.91	1.87
Mg-F_3	n.a.	1.94	1.90
Mg-O3β	n.a.	2.09	2.02
P_β-O1β	1.51	1.49	1.52
P_β-O2β	1.52	1.53	1.51
P_β-O3β	1.51	1.53	1.54
P_β-O3α	1.63	1.62	1.65
P_α-O1α	1.47	1.48	1.48
P_α-O2α	1.53	1.53	1.53
P_α-O3α	1.63	1.63	1.63
P_α-$O_{5'}$	1.60	1.60	1.60
**Bond Angles (degrees)**			
H-O(w3)-H	111.0	n.a	108.5
O3β-P_γ-O(w3)	174.7	n.a	n.a
O1γ-P_γ-O2γ	120.8	n.a	n.a
O1γ-P_γ-O3γ	121.9	n.a	n.a
O2γ-P_γ-O3γ	117.3	n.a	n.a
F_1-Mg-F_2	n.a	125.9	130.9
F_1-Mg-F_3	n.a	125.8	126.9
F_2-Mg-F_3	n.a	108.2	102.2
O1β-P_β-O2β	117.0	114.2	116.6
O1β-P_β-O3β	111.2	107.7	111.3
O1β-P_β-O3α	104.3	105.4	103.6
O2β-P_β-O3β	112.4	111.4	112.4
O2β-P_β-O3α	105.3	111.2	107.4
O3β-P_β-O3α	105.3	106.6	104.3
P_α-O3β-P_β	43.4	42.2	44.3
O1α-P_α-O2α	118.6	113.1	118.9
O1α-P_α-O3α	113.0	111.7	112.3
O1α-P_α-$O_{5'}$	109.2	108.2	109.1
O2α-P_α-O3α	106.1	111.9	105.8
O3α-P_α-$O_{5'}$	100.4	100.0	100.1
**Dihedral Angles (degrees)**			
O1γ-P_γ-O2γ-O3γ	176.2	n.a	n.a
F_1-Mg-F_2-F_3	n.a	-176.7	-176.9
O1γ-P_γ-P_β-O3β	137.8	n.a	n.a.
O1α-P_α-P_β-O1β	63.7	61.6	66.7

O1α-P_{α}-P_{β}-O2β	-51.0	-53.2	-49.1
O1α-P_{α}-P_{β}-O3β	-160.5	-164.3	-160.0
O2α-P_{α}-P_{β}-O1β	-176.3	-176.7	-172.8
O2α-P_{α}-P_{β}-O2β	69.0	53.2	71.3
O2α-P_{α}-P_{β}-O3β	-40.5	-164.3	-39.6
$O_{5'}$-P_{α}-P_{β}-O1β	-58.1	-58.8	-53.1
$O_{5'}$-P_{α}-P_{β}-O2β	-172.7	-173.6	-168.9
$O_{5'}$-P_{α}-P_{β}-O3β	77.8	75.3	80.2

Figure S1. **Michaelis complex structures for Ras superfamily GTPases with GTP analogs.** Analogs GPPNP (purple), GPPCP (gray) and GTPγS (orange) superposed by Cα alignment in PyMOL. Catalytic Mg (green) is shown in coordination to O2β, O1γ, Thr19 and Thr37 (RhoA numbering), and Lys18 is in coordination to O1β and O2γ (cyan dashes). Isolated water (colored as for corresponding GTP analog) is shown coordinating to O3γ, Thr37(CO), and variable backbone/side chain NH groups from Gln63. Hypervariable conformations of Gln63 side chains contrast with uniform locations of side chains of Lys18, Thr19, Thr37, and Gly62.

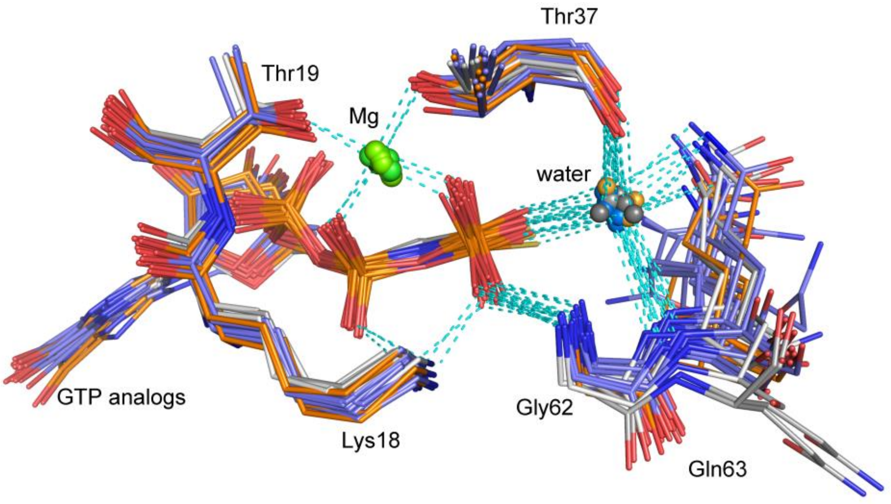

Figure S2. **Experimental ^{19}F NMR of the RhoA/GAP-GDP-MgF_3^- TSA complex in 100% H_2O (upper) and 100% D_2O (lower).** In order to suppress the broad free MgF^+ resonance (-155 ppm) by saturation transfer, continuous-wave ^{19}F radiation for the ^{19}F NMR experiments of the RhoA/GAP-GDP-MgF_3^- TSA complex was applied to the free fluoride resonance (-120 ppm) with a power level of 42 dB over a 1 s recycle delay. This completely removed signal overlap between the broad MgF^+ resonance and the F_2 resonance.

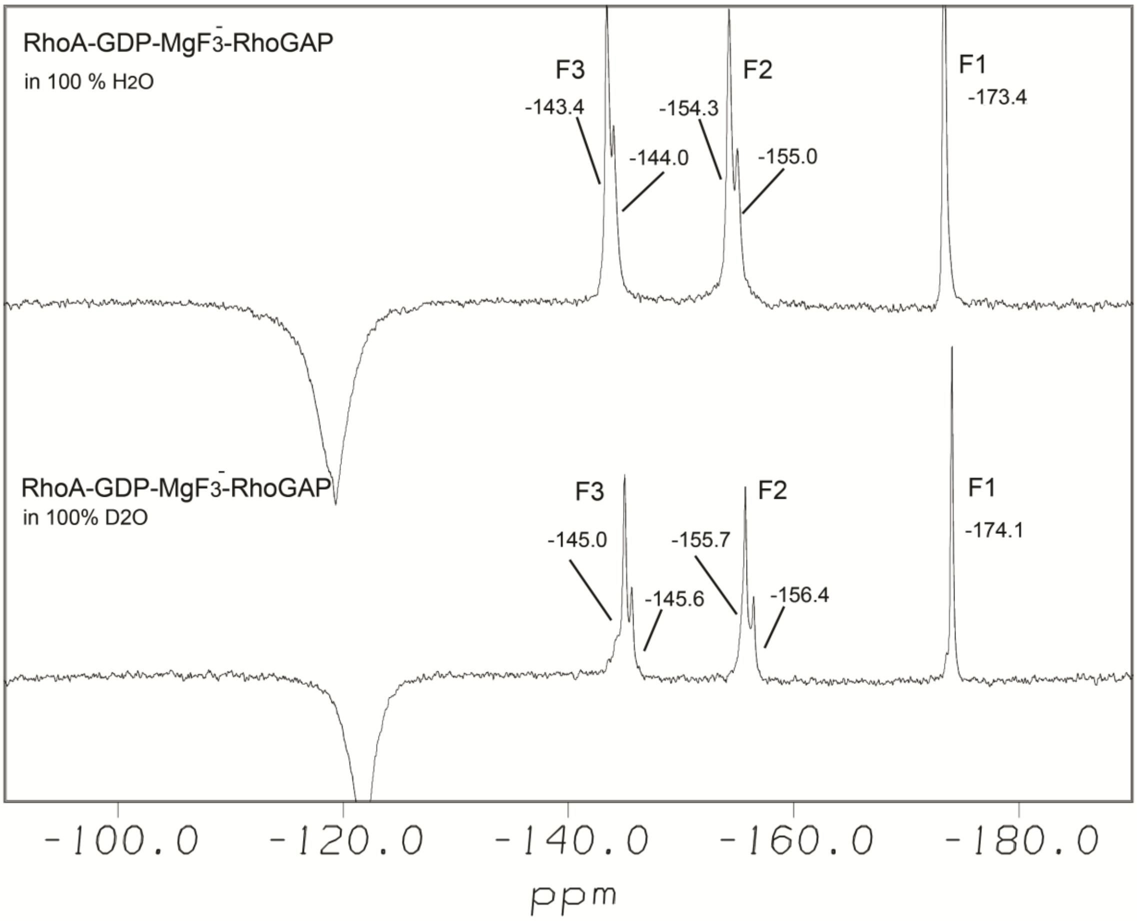

Figure S3. **Inclusion of Asp59 in the model is important for the correct computation of transition state.** Parent structure **1ow3** (gray sticks) has Ow2 (magenta sphere) at 4.31 Å separation from F2γ (light blue sphere) and coordinated to Asp59. An early computed structure (partially displayed for clarity, cyan sticks) based on **1ow3** but lacking Asp59 shows catalytic magnesium (green sphere) shifted and the O3β-Pγ bond rotated 20˚ to make Ow2 hydrogen bonded to O2γ (red spheres) at only 2.78 Å separation. Structures are aligned by pairing 11 terminal methyl groups in the computed structured with corresponding carbons in **1ow3,** hydrogens omitted for clarity).

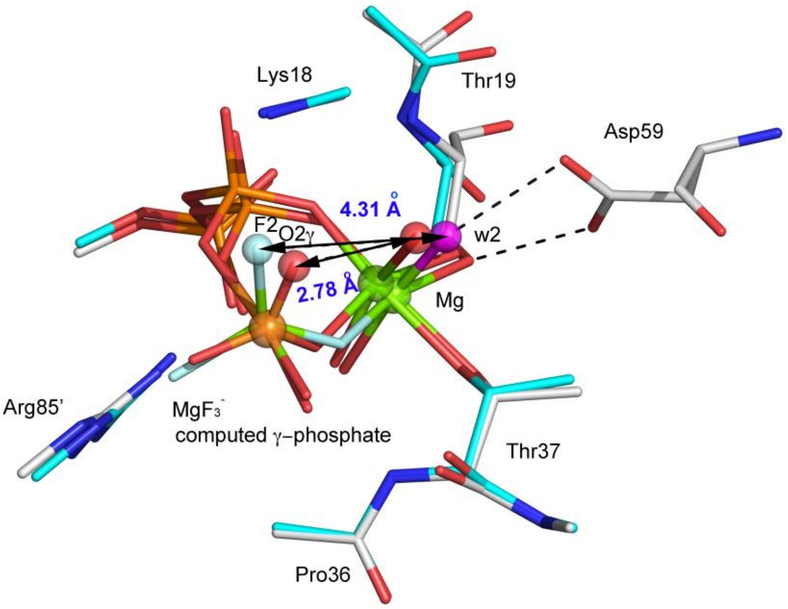

Figure S4. **The final computed TS structure for the RhoA/GAP-GTP complex.** The computed structure (green sticks, 91 heavy atoms) is overlaid on **1ow3** (silver sticks) with key 20 hydrogen bonds (black dashes). Ow3 is highlighted (red sphere) with catalytic magnesiums (magenta). The two structures are overlaid by aligning 10 terminal methyl groups (green spheres) on the corresponding carbons in **1ow3** (rmsd 0.001 Å). The complete atoms of the 17 participating amino acids are shown for **1ow3**; nonpolar hydrogens are omitted for clarity.

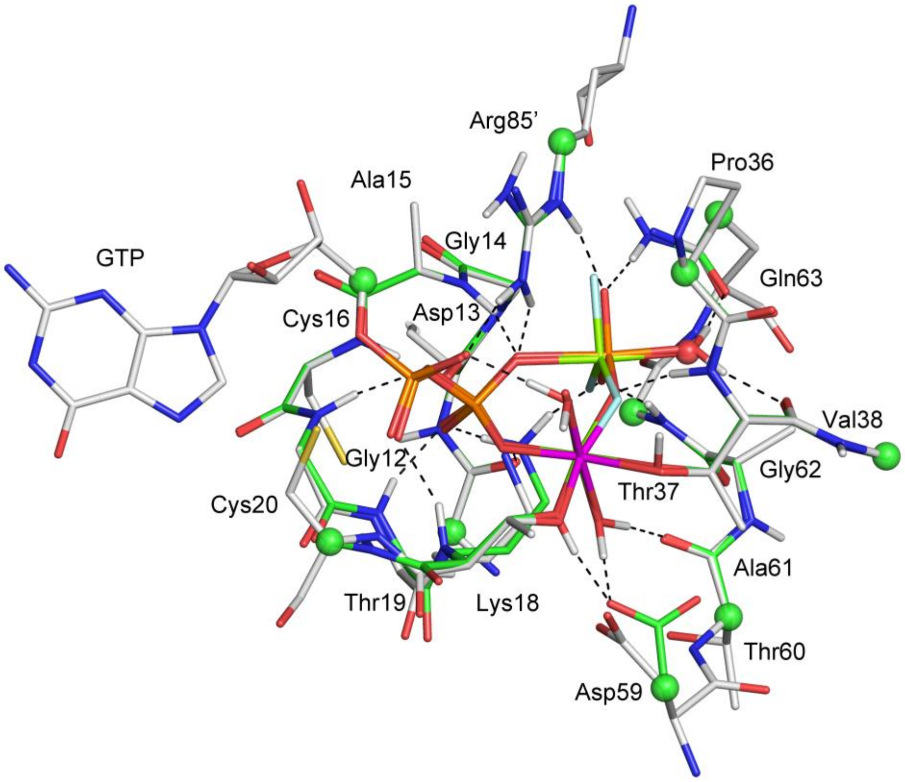

Figure S5. **Computed MgF_3^- TSA structure for RhoA/GAP-GDP-MgF_3^- complex.** Computed TSA structure for RhoA/GAP-GDP-MgF_3^- complex (cyan sticks) is overlaid on **1ow3** (gray sticks) with key 20 hydrogen bonds (black dashes) to the computed hydrogens (white sticks). The two structures are overlaid by aligning 11 terminal methyl groups on the corresponding carbons in **1ow3** (rmsd 0.05 Å). The complete amino acids are shown for **1ow3**; non-polar computed hydrogens are omitted for clarity.

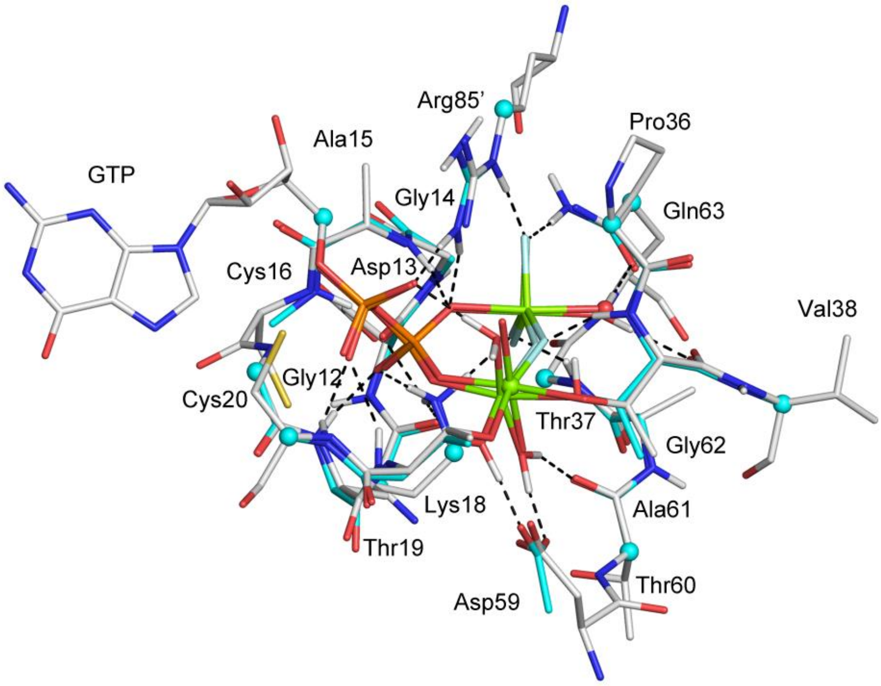

Figure S6. **Cross-eyed stereo view showing H-bonded Michaelis complex structures for 4 Ras superfamily GTPases with GTP.** GTPase structures (PDB: **1z0j**, **1n6l**, **2c5l**, **1wa5**) superposed by alignment of GTP (gray) on **1z0j** (1.3 Å resolution) and viewed down the bond Pγ–O3β). Catalytic Mg (green spheres) shown in coordination to O2β, O1γ, Thr19 and Thr37, Lys18, and two waters. The isolated water (dark gray) shown to be off-line with the Pγ–O3β bond. It coordinates O3γ, Thr37(CO), and Gln63(NH); in **1n6l**, the water also coordinates one of 2 conformations of the Gln63 side chain NH_2. Variable conformations of Gln63 (Leu63 for **1z0j**) side chains contrast with uniform orientations of side chains for Lys18, Thr19, Thr37, and Gly62.

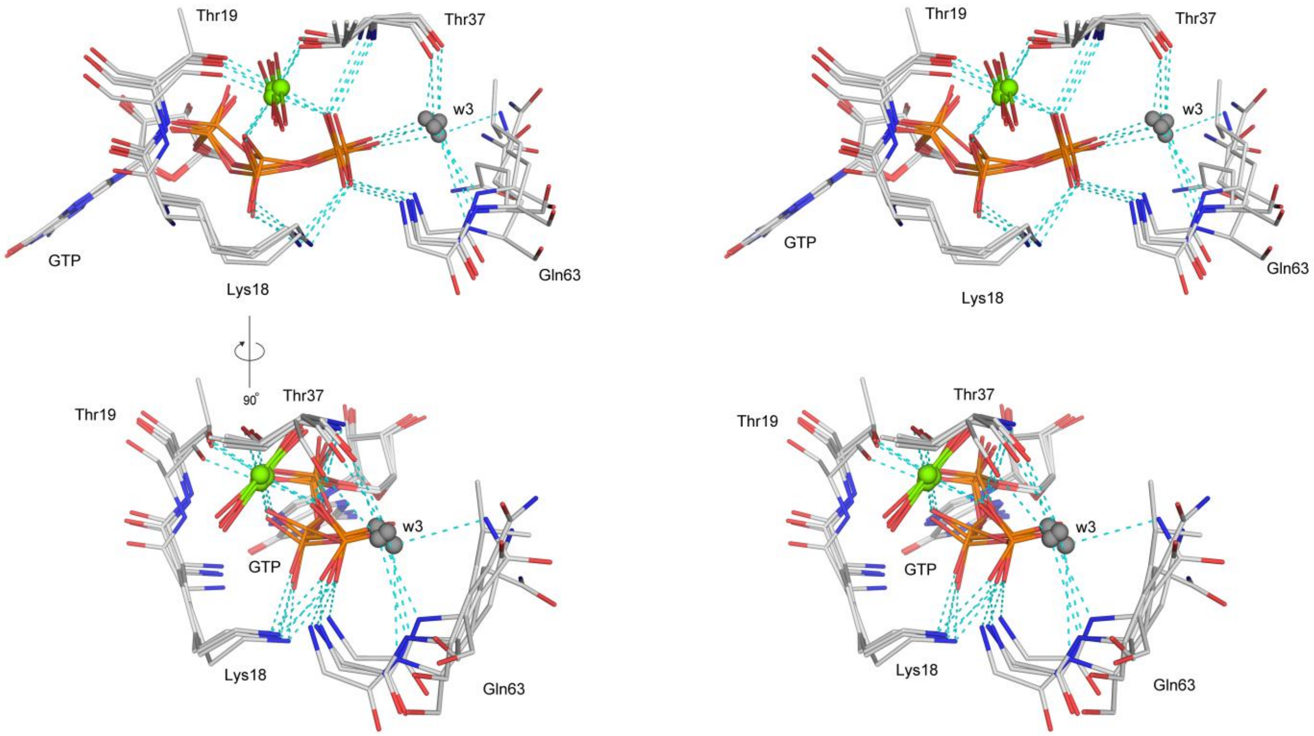

References:

[1] L. L. C. Schrodinger, The PyMOL Molecular Graphics System, Version 1.3r1 **2010**.

[2] K. Scheffzek, M. R. Ahmadian, W. Kabsch, L. Wiesmuller, A. Lautwein, F. Schmitz, A. Wittinghofer, *Science* **1997**, *277*, 333-338.

[3] D. L. Graham, J. F. Eccleston, C. W. Chung, P. N. Lowe, *Biochemistry* **1999**, *38*, 14981-14987.

[4] D. L. Graham, P. N. Lowe, G. W. Grime, M. Marsh, K. Rittinger, S. J. Smerdon, S. J. Gamblin, J. F. Eccleston, *Chem. Biol.* **2002**, *9*, 375-381.

[5] N. J. Baxter, G. M. Blackburn, J. P. Marston, A. M. Hounslow, M. J. Cliff, W. Bermel, N. H. Williams, F. Hollfelder, D. E. Wemmer, J. P. Waltho, *J. Am. Chem. Soc.* **2008**, *130*, 3952-3958.

[6] a) N. J. Baxter, L. F. Olguin, M. Golicnik, G. Feng, A. M. Hounslow, W. Bermel, G. M. Blackburn, F. Hollfelder, J. P. Waltho, N. H. Williams, *Proc. Natl. Acad. Sci. USA* **2006**, *103*, 14732-14737; b) N. J. Baxter, A. M. Hounslow, M. W. Bowler, N. H. Williams, G. M. Blackburn, J. P. Waltho, *J. Am. Chem. Soc.* **2009**, *131*, 16334-16335; c) N. J. Baxter, M. W. Bowler, T. Alizadeh, M. J. Cliff, A. M. Hounslow, B. Wu, D. B. Berkowitz, N. H. Williams, G. M. Blackburn, J. P. Waltho, *Proc. Natl. Acad. Sci. USA* **2010**, *107*, 4555-4560; d) M. J. Cliff, M. W. Bowler, A. Varga, J. P. Marston, J. Szabo, A. M. Hounslow, N. J. Baxter, G. M. Blackburn, M. Vas, J. P. Waltho, *J. Am. Chem. Soc.* **2010**, *132*, 6507-6516; e) J. L. Griffin, M. W. Bowler, N. J. Baxter, K. N. Leigh, H. R. W. Dannatt, A. M. Hounslow, G. M. Blackburn, C. E. Webster, M. J. Cliff, J. P. Waltho, *Proc. Natl. Acad. Sci. USA* **2012**, *109*, 6910-6915; f) Y. Jin, M. J. Cliff, N. J. Baxter, H. R. W. Dannatt, A. M. Hounslow, M. W. Bowler, G. M. Blackburn, J. P. Waltho, *Angew. Chem. Int. Ed.* **2012**, *51*, 12242-12245; *Angew. Chem.* **2012**, 124, 12408-12411; g) Y. Jin, D. Bhattasali, E. Pellegrini, S. M. Forget, N. J. Baxter, M. J. Cliff, M. W. Bowler, D. L. Jakeman, G. M. Blackburn, J. P. Waltho, *Proc. Natl. Acad. Sci. USA* **2014**, *111*, 12384-12389.

[7] Y. Zhao, D. Truhlar, *Theor. Chem. Acc.* **2008**, *120*, 215-241.

[8] a) T. H. Dunning, *J. Chem. Phys.* **1989**, *90*, 1007-1023; b) D. E. Woon, T. H. Dunning, *J. Chem. Phys.* **1993**, *98*, 1358-1371.

[9] X. Li, M. J. Frisch, *J. Chem.Theory Comput.* **2006**, *2*, 835-839.

[10] M. J. Frisch, G. W. Trucks, H. B. Schlegel, G. E. Scuseria, M. A. Robb, J. R. Cheeseman, G. Scalmani, V. Barone, B. Mennucci, G. A. Petersson, H. Nakatsuji, M. Caricato, X. Li, H. P. Hratchian, A. F. Izmaylov, J. Bloino, G. Zheng, J. L. Sonnenberg, M. Hada, M. Ehara, K. Toyota, R. Fukuda, J. Hasegawa, M. Ishida, T. Nakajima, Y. Honda, O. Kitao, H. Nakai, T. Vreven, J. A. Montgomery Jr., J. E. Peralta, F. Ogliaro, M. J. Bearpark, J. Heyd, E. N. Brothers, K. N. Kudin, V. N. Staroverov, R. Kobayashi, J. Normand, K. Raghavachari, A. P. Rendell, J. C. Burant, S. S. Iyengar, J. Tomasi, M. Cossi, N. Rega, N. J. Millam, M. Klene, J. E. Knox, J. B. Cross, V. Bakken, C. Adamo, J. Jaramillo, R. Gomperts, R. E. Stratmann, O. Yazyev, A. J. Austin, R. Cammi, C. Pomelli, J. W. Ochterski, R. L. Martin, K. Morokuma, V. G. Zakrzewski, G. A. Voth, P. Salvador, J. J. Dannenberg, S. Dapprich, A. D. Daniels, Ö. Farkas, J. B. Foresman, J. V. Ortiz, J. Cioslowski, D. J. Fox, Gaussian, Inc., Wallingford, CT, USA, **2009**.

[11] R. A. Kendall, T. H. Dunning, R. J. Harrison, *J. Chem. Phys.* **1992**, *96*, 6796-6806.

[12] a) C. Trindle, D. Shillady, *Electronic Structure Modeling*, CRC Press, Boca Raton, **2008**, pp. 350-352; b) T. Helgaker, P. Jorgensen, *J. Chem. Phys.* **1991**, *95*, 2595-2601.

[13] C. Cramer, *Essentials of Computational Chemistry*, Wiley, New York, **2004**, 344-349.

[14] a) J. Reuben, R. Y. Shvo, A. Demiel, *J. Amer. Chem. Soc.* **1965**, *87*, 3995-3996. (1,1,2-trifluoroethene)

b) R. A. Beaudet, J. D. Baldeschwieler, *J. Mol. Spectrosc.* **1962**, *9*, 30-41. (1,1-difluoro-2-methylethene)

c) F. A. Bovey, E. W. Anderson, F. P. Hood, R. L. Kornegay, *J. Chem. Phys.* **1964**, *40*, 3099-3109. (Fluorocyclohexane; difference in axial vs. equatorial peaks)

d) J. A. Martin, *Tetrahedon Lett.* **1966**, *7*, 2879-2882. (Fluorocyclohexane; difference in axial vs. equatorial peaks)

e) S. L. Spassov, D. L. Griffith, E. S. Glazer, N. Kuppswamy, J. D. Roberts, *J. Amer. Chem. Soc.* **1967**, *89*, 89-94. (1,1-difluorocyclohexane; comparison of axial vs equatorial peaks)

f) S. L. Spassov, D. L. Griffith, E. S. Glazer, N. Kuppswamy, J. D. Roberts, *J. Amer. Chem. Soc.* **1967**, *89*, 89-94. (1,1,2,2-tetrafluorocyclohexane; comparison of axial vs. equatorial peaks).

g) J. W. Emsley, L. Phillips, *Prog. Nucl. Magn. Reson. Spectrosc.* **1971**, *7*, 281. (1,2,3,4,4,5,5,6-octafluorocyclohexa-1,2-diene; 2 out of 3 possible peaks compared)

h) J. W. Emsley, L. Phillips, *Prog. Nucl. Magn. Reson. Spectrosc.* **1971**, *7*, 281. (1,2,3,4,4,5,5,6-octafluorocyclohexa-1,2-diene; comparison of another 2 out of 3 possible peaks)

i) J. W. Emsley, L. Phillips, *Prog. Nucl. Magn. Reson. Spectrosc.* **1971**, *7*, 281. (1,2,2,3,4,5,5,6-octafluorocyclohexa-1,3-diene).

j) S. G. Campbell, *Spectrochim. Acta. A. Mol. Biomol. Spectrosc.* **1967**, 23, 2119-2125. (3,4,5,6-tetrafluorocyclo-1-hexene)

k) G. L. Caldow, *Mol. Phys.* **1966**, 11, 71-83. (1,2,3,4,5-pentafluorobenzene; comparison of 2 out of 3 possible *ortho-, meta-, para*-peaks)

l) J. W. Emsley, L. Phillips, **1971**, *7*, 392. (1,2,3,4,5-pentafluorobenzene; comparison of 2 out of 3 possible *ortho-, meta-, para*-peaks)

m) G. L. Caldow, *Mol. Phys.* **1966**, *11*, 71-83. (1,2,3,4,5-pentafluorobenzene; comparison of other 2 out of 3 possible *ortho-, meta-, para*-peaks)

n) J. W. Emsley, L. Phillips, *Prog. Nucl. Magn. Reson. Spectrosc.* **1971**, *7*, 392. (1,2,3,4,5-pentafluorobenzene; comparison of other 2 out of 3 possible *ortho-, meta-, para*-peaks)

o) J. Homer, L. F. Thomas, *J. Chem. Soc. B.* **1966**, 141-144. (1-amino-2,3,4,5,6-pentafluorobenzene; comparison of 2 out of 3 possible *ortho-, meta-, para*-peaks)

p) J. Homer, L. F. Thomas, *J. Chem. Soc. B.* **1966**, 141-144. (1-amino-2,3,4,5,6-pentafluorobenzene; comparison of another 2 out of 3 possible *ortho-, meta-, para*-peaks)

q) R. C. Golike, *J. Mol. Spectrosc.* **1965**, *16*, 214-215. (2,2,3,3-tetrafluoro-1-propanol)

r) M. Lustig, A. R. Pitochelli, J. K. Ruff, *J. Amer. Chem. Soc.* **1967**, *89*, 2841-2843. (1,1,2,2-tetrafluoro-2-amino-1-ethanol)

s) D. D. Elleman, L. C. Brown, D. Williams, *J. Mol. Spectrosc.* **1961**, *7*, 322-340. (1,1,2,2,3,3-hexafluoropropane)

t) J. H. Prager, P. G. Thompson, *J. Amer. Chem. Soc.* **1965**, *87*, 230-238. (1,1,2,2,3,3,3-heptafluoro-1-propanol)

u) R. A. Mitsch, *J. Amer. Chem. Soc.* ***1965***, *87*, 328-333. (perfluoro-2,3-diaminopropane)

v) M. H. Kaufmann, J. D. Braun, J. G. Shdo, (1967) Synthesis of terminal perfluoromethylene olefins. *J. Org. Chem.* **1967**, *32*, 2749-2751. (1,1-difluoro-2-trifluoromethyl-2-ethylethene)

w) J. W. Emsley, L. Phillips, *Prog. Nucl. Magn. Reson. Spectrosc.* **1971**, p.238. (1,1-difluoro-2-trifluoromethyl-2-ethylethene)

[15] N. J. R. van Eikema Hommes, Clark T, *J. Mol. Model.* **2005**, *11*, 175-185.

[16] a) H. te Heesen, K. Gerwert, J. Schlitter, FEBS Lett. **2007**, *581*, 5677-5684; b) S. C. Kamerlin, P. K. Sharma, R. B. Prasad, A. Warshel, *Q. Rev. Biophys.* **2013**, *46*, 1-132.

[17] a) A. S. Mildvan, *Proteins* **1997**, *29*, 401-416; b) H. te Heesen, K. Gerwert, J. Schlitter, *FEBS Lett.* **2007**, *581*, 5677-5684; c) A. Wittinghofer, *Trends Biochem. Sci.* **2006**, *31*, 20-23.

[18] T. M. Glennon, J. Villà, A. Warshel, *Biochemistry* **2000**, *39*, 9641-9651.

[19] B. L. Grigorenko, A. V. Nemukhin, R. E. Cachau, I. A. Topol, S. K. Burt, *J. Mol. Model.* **2005**, *11*, 503-508.

[20] J. K. Lassila, J. G. Zalatan, D. Herschlag, *Annu. Rev. Biochem.* **2011**, *80*, 669-702.

[21] D. Roy, K. Todd, M.John. *Semichem Inc.*, Shawnee Mission, KS, **2009**.
